# Retraction: Iron Supplementation Decreases Severity of Allergic Inflammation in Murine Lung

**DOI:** 10.1371/journal.pone.0155387

**Published:** 2016-05-09

**Authors:** Laura P. Hale, Erin Potts Kant, Paula K. Greer, W. Michael Foster

The authors of the published article have identified concerns about the reliability of a subset of the reported data, specifically the data underlying Figures 1 and 2. Potential discrepancies have been discovered between the machine-generated raw data and the data for publication provided by the murine pulmonary function laboratory, and the authors have become aware of potential issues in methodology that could make the data unreliable. Figures 3 and 4 are not affected.

In light of the concerns identified, the authors retract this publication.
